# Potentiation of the cytotoxicity of thymidylate synthase (TS) inhibitors by dipyridamole analogues with reduced **α**_1_-acid glycoprotein binding

**DOI:** 10.1038/sj.bjc.6690591

**Published:** 1999-08

**Authors:** N J Curtin, K J Bowman, R N Turner, B Huang, P J Loughlin, A H Calvert, B T Golding, R J Griffin, D R Newell

**Affiliations:** Cancer Research Unit and Department of Chemistry, University of Newcastle upon Tyne, Framlington Place, Newcastle upon Tyne, NE2 4HH, UK

**Keywords:** nucleoside transport inhibition, dipyridamole analogues, α_1_-acid glycoprotein, thymidine rescue, nolatrexed cytotoxicity, 5-fluorouracil cytotoxicity

## Abstract

Dipyridamole has been shown to enhance the in vitro activity of antimetabolite anticancer drugs through the inhibition of nucleoside transport. However, the clinical potential of dipyridamole has not been realized because of the avid binding of the drug to the plasma protein α_1_-acid glycoprotein (AGP). Dipyridamole analogues that retain potent nucleoside transport inhibitory activity in the presence of AGP are described and their ability to enhance the growth inhibitory and cytotoxic effects of thymidylate synthase (TS) inhibitors has been evaluated. Three dipyridamole analogues (NU3026, NU3059 and NU3060) were shown to enhance the growth inhibitory activity of the TS inhibitor CB3717 and block thymidine rescue in L1210 cells. The extent of potentiation at a fixed analogue concentration (10 μM) was related to the potency of inhibition of thymidine uptake. A further analogue, NU3076, was identified, which was more potent than dipyridamole with a *K*_i_ value for inhibition of thymidine uptake of 0.1 μM compared to 0.28 μM for dipyridamole. In marked contrast to dipyridamole, inhibition of thymidine uptake by NU3076 was not significantly affected by the presence of AGP (5 mg ml^−1^). NU3076 and dipyridamole produced equivalent potentiation of the cytotoxicity of the non-classical antifolate TS inhibitor, nolatrexed, in L1210 cells with both compounds significantly reducing the LC_90_ by > threefold in the absence of salvageable thymidine. Thymidine rescue of L1210 cells from nolatrexed cytotoxicity was partially blocked by both 1 μM NU3076 and 1 μM dipyridamole. NU3076 also caused a significant potentiation of FU cytotoxicity in L1210 cells. These studies demonstrate that nucleoside transport inhibition can be maintained in the absence of AGP binding with the dipyridamole pharmacophore and that such analogues can enhance the cytotoxicity of TS inhibitors. © 1999 Cancer Research Campaign


*British Journal of Cancer* (1999) **80**(11), 1738–1746
© 1999 Cancer Research Campaign
Article no. bjoc.1999.0591

### Potentiation of the cytotoxicity of thymidylate synthase (TS) inhibitors by dipyridamole analogues with reduced $\alpha_1$-acid glycoprotein binding

**NJ Curtin[1], KJ Bowman[1], RN Turner[1], B Huang[2], PJ Loughlin[2], AH Calvert[1], BT Golding[2], RJ Griffin[2] and DR Newell[1]**

[1]Cancer Research Unit and [2]Department of Chemistry, University of Newcastle upon Tyne, Medical School, Framlington Place, Newcastle upon Tyne NE2 4HH, UK

**Summary** Dipyridamole has been shown to enhance the in vitro activity of antimetabolite anticancer drugs through the inhibition of nucleoside transport. However, the clinical potential of dipyridamole has not been realized because of the avid binding of the drug to the plasma protein $\alpha_1$-acid glycoprotein (AGP). Dipyridamole analogues that retain potent nucleoside transport inhibitory activity in the presence of AGP are described and their ability to enhance the growth inhibitory and cytotoxic effects of thymidylate synthase (TS) inhibitors has been evaluated. Three dipyridamole analogues (NU3026, NU3059 and NU3060) were shown to enhance the growth inhibitory activity of the TS inhibitor CB3717 and block thymidine rescue in L1210 cells. The extent of potentiation at a fixed analogue concentration (10 μM) was related to the potency of inhibition of thymidine uptake. A further analogue, NU3076, was identified, which was more potent than dipyridamole with a $K_i$ value for inhibition of thymidine uptake of 0.1 μM compared to 0.28 μM for dipyridamole. In marked contrast to dipyridamole, inhibition of thymidine uptake by NU3076 was not significantly affected by the presence of AGP (5 mg ml$^{-1}$). NU3076 and dipyridamole produced equivalent potentiation of the cytotoxicity of the non-classical antifolate TS inhibitor, nolatrexed, in L1210 cells with both compounds significantly reducing the $LC_{90}$ by > threefold in the absence of salvageable thymidine. Thymidine rescue of L1210 cells from nolatrexed cytotoxicity was partially blocked by both 1 μM NU3076 and 1 μM dipyridamole. NU3076 also caused a significant potentiation of FU cytotoxicity in L1210 cells. These studies demonstrate that nucleoside transport inhibition can be maintained in the absence of AGP binding with the dipyridamole pharmacophore and that such analogues can enhance the cytotoxicity of TS inhibitors.

**Keywords:** nucleoside transport inhibition; dipyridamole analogues; $\alpha_1$-acid glycoprotein; thymidine rescue; nolatrexed cytotoxicity; 5-fluorouracil cytotoxicity

Antimetabolite inhibitors of de novo purine and pyrimidine synthesis are used extensively in cancer chemotherapeutic regimes (Schilsky, 1992). In particular, inhibition of thymidylate synthase (TS) is an attractive target for antimetabolite chemotherapy because thymine is the only nucleobase found exclusively in DNA and TS is a key enzyme, possibly the rate-limiting step, in DNA synthesis. One locus of action of 5-fluorouracil (5-FU), which is widely used for the treatment of colorectal, breast and head-and-neck cancer, is TS inhibition following its intracellular conversion to 5-FdUMP. CB3717 ($N^{10}$-propargyl-5,8-dideazafolate) was the first selective antifolate inhibitor of TS to be developed (Jones et al, 1981), and in early clinical trials with CB3717 promising therapeutic activity was reported (Calvert et al, 1986). More recently, a number of antifolate TS inhibitors have been developed, i.e. raltitrexed, nolatrexed, MTA, AG 331, BW 1843 U89 and ZD9331, and activity has been observed against a broad range of tumours in a number of clinical trials with these agents (Rustum et al, 1997).

Inhibition of TS results in the depletion of intracellular dTTP and, as a result of elevated dUMP levels, an accummulation of dUTP. This nucleotide pool imbalance contributes to DNA strand breakage and cell death (Curtin et al, 1991). Salvage of extracellular thymidine, mediated by nucleoside transport and subsequent phosphorylation by thymidine kinase, restores thymidylate pools and hence limits the activity of TS inhibitors (Jackman et al, 1984). A number of factors may promote utilization of the salvage pathway by tumours, for example, Weber (1983) has demonstrated that the activities of the enzymes involved in nucleoside salvage can exceed those of the de novo pathway for nucleotide synthesis in a variety of tumours. Furthermore, resistance to antimetabolities mediated by nucleoside salvage may increase with malignancy (Fox et al, 1991; Kinsella and Harran, 1991). In addition, release of nucleic acids from dead cells may result in high local concentrations of salvageable nucleosides and bases in tumours.

Salvage of pre-formed nucleosides is dependent on uptake across the plasma membrane into the cell. At physiological nucleoside concentrations the principal mode of nucleoside uptake is via the *es* and *ei* equilibrative carriers, described as such on the basis of their sensitivity (*es*) or insensitivity (*ei*) to the transport inhibitor nitrobenzylmercaptopurine riboside (NBMPR). The cardiovascular and antiplatelet drug, dipyridamole, inhibits both *es* and *ei* carriers, thereby blocking the uptake of nucleosides for salvage. Dipyridamole has been used successfully in vitro, and to a limited extent in animal studies, to increase the activity of a range of antimetabolites (reviewed in Goel and Howell, 1992). In addition to preventing thymidine uptake, dipyridamole also enhances

*Received 4 November 1998*
*Revised 26 January 1999*
*Accepted 2 February 1999*

*Correspondence to:* NJ Curtin

**Table 1** Inhibition of thymidine uptake by novel dipyridamole analogues in the presence or absence of 5 mg $ml^{-1}$ AGP

| Compound | Structure | Inhibitor concentration | | | |
|---|---|---|---|---|---|
| | | **1 μM** | **10 μM** | **10 μM + AGP** | **$IC_{50}{}^{b}$ (μM)** |
| Dipyridamole<br><br>2,6-*bis*-(diethanolamino)-4,8-di-piperidino-pyrimidopyrimidine |  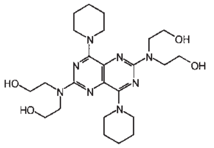 | 80 ± 8[a]<br>(26) | 99 ± 2<br>(84) | 13 ± 12<br>(27)[d,e] | 0.36,<br>0.37 |
| NU3026 (*RR*)[c]<br><br>2,6-di-(2,2-dimethyl-1,3-dioxolan-4-yl)-methoxy-4,8-di-piperidinopyrimido-pyrimidine |  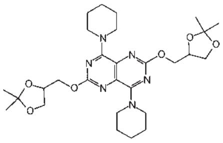 | 47 ± 5<br>(6) | 87 ± 8<br>(15) | 62 ± 12<br>(8)[d] | 1.2,<br>1.1 |
| NU3059<br><br>2,6-bis-(2,3-dimethoxypropoxy)-4,8-di-piperidino-pyrimidopyrimidine |  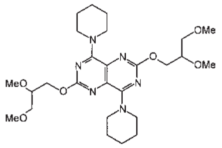 | 38 ± 17<br>(15) | 87 ± 7<br>(26) | 67 ± 17<br>(5)[d] | 1.9 ± 1<br>(5) |
| NU3060<br><br>2,6-bis[*N*,*N*-di(2-methoxy)ethyl]-4,8-di-piperidino-pyrimidopyrimidine |  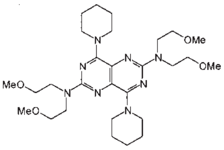 | 29 ± 15<br>(12) | 86 ± 11<br>(27) | 43 ± 8<br>(9)[d] | 2.1 ± 0.7<br>(3) |

Data are individual values or mean ± s.d. for the number of observations given in parenthesis. [a]Data are the % inhibition of thymidine uptake obtained in the absence or presence of 5 mg $ml^{-1}$ AGP. [b]$IC_{50}$ values for inhibition of thymidine uptake in the absence of AGP (calculated from a point-to-point curve using GraphPad Prism™ software). [c]The (*S,S*)- and (*R,S*)-isomers showed similar potency. [d]Significantly different from value obtained in the absence of AGP (unpaired Student's *t*-test). [e]Not significantly different from zero.

the cytotoxicity of TS inhibitors by preventing the efflux of deoxyuridine, leading to a greater accumulation of dUTP (Curtin et al, 1991). Similarly, potentiation of 5-FU cytotoxicity by dipyridamole may be mediated not only through inhibition of the uptake of thymidine for salvage, but also by the inhibition of deoxyuridine and fluorodeoxyuridine efflux, leading to higher intracellular dUMP, FdUMP and FdUTP concentrations (Grem and Fisher, 1985, 1989).

Despite these promising in vitro data with antimetabolite/dipyridamole combinations, improved therapy in patients has not been achieved. For example, although some phase I studies suggested an enhancement of 5-FU and methotrexate activity by combination with dipyridamole (reviewed in Schmoll et al, 1990), in phase II and III trials the combination with dipyridamole did not improve the antitumour activity of methotrexate or 5-FU (Wadler et al, 1987; Köhne-Wömpner et al, 1989). The discrepancy between the clinical and preclinical results obtained with dipyridamole may be due to the avid binding of the drug to $\alpha_1$-acid glycoprotein (AGP), (Mahony et al, 1982), an acute phase protein that is elevated in the plasma of cancer patients. In two studies of patients receiving the maximum tolerated dose of dipyridamole peak total plasma concentrations of dipyridamole of 12 μM and 16 μM were achieved (Willson et al, 1988; Budd et al, 1990), concentrations which should have been sufficient to prevent nucleoside transport on the basis of in vitro data; however, the free plasma concentration of dipyridamole was only 27 nM and 38 nM in these studies, which is insufficient to enhance antimetabolite activity in vitro. In agreement with the clinical data, physiological levels of AGP have also been shown to prevent the potentiation of CB3717 growth inhibition by dipyridamole in vitro (Curtin et al, 1989).

On the basis of in vitro data it should be possible to use nucleoside transport inhibitors to enhance the clinical activity of TS inhibitors, provided the limitations of AGP binding can be overcome. The aim of the studies described here was to investigate the effect of novel dipyridamole analogues on the in vitro growth inhibitory activity of TS. Two antifolate TS inhibitors were selected for study: the classical antifolate, CB3717 ($N^{10}$-propargyl-5,8-dideazafolic acid), and the non-classical antifolate TS inhibitor, nolatrexed (AG337, Thymitaq, 3,4-dihydro-2-amino-6-methyl-4-oxo-5-(4-pyridylthio)-quinazoline dihydrochloride) (Webber et al, 1996). In addition, potentiation of the cytotoxicity of 5-FU was investigated. Data for selected dipyridamole analogues that are potent inhibitors of thymidine transport, even in the presence of AGP, are presented. Studies with NU3026, NU3059 and NU3060 demonstrated that the degree of enhancement of growth inhibition by TS inhibitors was dependent on the potency of the compounds as inhibitors of thymidine transport. A further inhibitor, NU3076, was identified, which was more potent

© 1999 Cancer Research Campaign 

than dipyridamole, active in the presence of AGP and able to potentiate both nolatrexed and 5-FU cytotoxicity.

### MATERIALS AND METHODS

### Drugs

CB3717 (a gift from Zeneca Pharmaceuticals, Alderley Park, UK) was dissolved in 0.15 M sodium hydrogen carbonate and stored at 4°C. Nolatrexed (a gift from Agouron Pharmaceuticals Inc, San Diego, USA), 5-FU and dipyridamole (Sigma Chemical Co, Poole, UK), NU3026, NU3059, NU3060 and NU3076 (synthesized in the Department of Chemistry, University of Newcastle upon Tyne, UK; for structures see Tables 1 and 2) were dissolved in dimethyl sulphoxide (DMSO) and stored at –20°C. All other chemicals were obtained from Sigma unless stated otherwise.

### Cell culture

Murine leukaemia L1210 cells were used because their nucleoside transport characteristics have been well defined (Crawford et al, 1990). Cells were maintained as exponentially growing cultures ($< 8 \times 10^5$ cells $ml^{-1}$) in RPMI-1640 medium supplemented with 10% (v/v) fetal calf serum (Sigma), at 37°C in an atmosphere of 5% carbon dioxide in air. The cell doubling time was approximately 12 h. For growth inhibition and cytotoxicity assays, cells were adapted to grow in RPMI medium supplemented with 10% (v/v) dialysed serum (dialysed for 24 h at 4°C against two changes of 9 volumes of phosphate-buffered saline (PBS) containing 1 g activated charcoal $L^{-1}$ and a further two changes of 9 volumes of PBS) to remove thymidine. Once transferred to medium containing dialysed serum cells were passaged for at least 4 weeks prior to cytotoxicity assays, when the cell doubling time was also approximately 12 h. Cells were routinely tested to exclude mycoplasma contamination (Chen, 1977).

### Nucleoside transport inhibition assay

Thymidine uptake was assayed in L1210 cells using a modification of the rapid mixing technique of Wohlheuter et al (1978). The uptake of 100 μM thymidine by $10^6$ cells was followed at 2-second intervals over a 12-second time course in the presence or absence of inhibitor in 1% or 5% (v/v) DMSO. In experiments to determine the effect of AGP, the uptake of thymidine was also measured in the presence of 5 mg $ml^{-1}$ human AGP (Sigma), which is the upper limit of plasma AGP concentrations in cancer patients, i.e. approximately 125 μM (Kremer et al, 1988).

Exponentially growing L1210 cells were washed with and resuspended in ice-cold transport buffer (130 mM sodium chloride, 5 mM potassium chloride, 1 mM magnesium chloride, 5 mM $NaH_2PO_4$, 10 mM glucose and 10 mM HEPES pH 7.4) at $2–4 \times 10^7$ cells $ml^{-1}$. Cells were left on ice for 30 min prior to starting the assay and then diluted further in transport buffer ± AGP (7.5 mg $ml^{-1}$) containing 1.5% or 7.5% (v/v) DMSO (± inhibitor at 1.5 × the final concentration) for 5 min at 21°C. The suspension was mixed and 100 μl was layered onto 150 μl silicone oil (9:11 Dow Corning 556 (Sp.Gr. 0.98): Dow Corning 550 (Sp. Gr. 1.068), final Sp. Gr. 1.028) overlaying 50 μl 3 M potassium hydroxide (KOH), in six replicate 0.5 ml microfuge tubes. Transport was initiated by adding 50 μl of 300 μM thymidine in transport buffer, containing 25 μCi $ml^{-1}$ [methyl-$^3$H]thymidine (Sp. Act. 1.5–2.2 TBq $mmol^{-1}$; Amersham, UK) and 2 μCi $ml^{-1}$ [U-$^{14}$C]sucrose (17–27 GBq $mmol^{-1}$; Amersham), to each tube in turn at 1-second intervals, giving final concentrations of: $10^6$ cells in transport buffer± 5 mg $ml^{-1}$ AGP, 100 μM thymidine and 1% or 5% (v/v) DMSO± inhibitor. Transport was stopped by the addition of 50 μl of400 μM dipyridamole at 1-second intervals to the tubes in reverse order. The tubes were immediately centrifuged at 12 000***g*** for 2 min at room temperature causing the cells to pass through the oil and into the KOH. [$^{14}$C]Sucrose, which does not enter cells with intact plasma membranes, was included to allow calculation of the amount of extracellular fluid passing through the oil, and hence the amount of non-transported contaminating [$^3$H]thymidine in the extracellular space. Cell number and viability in each assay was determined by haemocytometer counting using trypan blue exclusion. After centrifugation the tubes were capped and cut in the oil layer such that the bottom portion (cells solubilized in KOH) fell into a 20 ml scintillation vial. One millilitre of 0.25 M acetic acid was injected into the tube to disperse and neutralize the KOH-solubilized cells and following the addition of 10 ml of Optiphase HiSafe scintillant (Fischer Chemicals, Loughborough, UK) the radioactivity in the cells was determined by a dual label assay using an LKB-Wallac S1410 β-counter (Wallac (UK), Milton Keynes, UK). The rate of thymidine uptake in the presence and absence of inhibitor was calculated by unweighted linear regression analysis of thymidine uptake (pmol $10^{-6}$ cells) vs time (seconds) using GraphPad PRISM™ software (GraphPad Software Inc. San Diego, CA, USA).

**Table 2** Comparison of the potentiation of CB3717-induced growth inhibition by NU3026, NU3059, NU3060 and dipyridamole in L1210 cells in the presence or absence of thymidine

| ± Nucleoside transport inhibitor | % Control cell growth + 3 μM CB3717 | | % Control cell growth + 10 μM CB3717 | |
|---|---|---|---|---|
| | no TdR | + 1 μM TdR | no TdR | + 1 μM TdR |
| No inhibitor | 40 | 104 | 5 | 107 |
| 10 μM Dipyridamole | 4 | 4 | 0 | 1 |
| 10 μM NU3059 | 5 | 10 | 0 | 7 |
| 10 μM NU3026 | 8 | 26 | 1 | 20 |
| 10 μM NU3060 | 29 | 59 | 3 | 37 |

Cell growth was determined after 48 hour exposure to 3 μM or 10 μM CB3717 with or without 1 μM thymidine supplementation in the presence or absence of 10 μM dipyridamole, NU3026, NU3059 or NU3060. Data, normalized by comparison with cell growth in the presence of 10 μM dipyridamole, NU3026, NU3059 or NU3060 alone, are the mean of three independent observations. Standard deviations (not shown) were < 15% of the mean in each case.

### Inhibition kinetics

Dipyridamole and NU3076 inhibition kinetics were investigated by modifying the transport assay to measure the uptake of thymidine at concentrations ranging from 100 to 1500 μM in the presence or absence of 0.3 μM dipyridamole or NU3076. $K_t$ and $T_{max}$ values were calculated using the equation describing a one-site binding hyperbola, fitted using GraphPad PRISM software. The $K_i$ values for dipyridamole and NU3076 were determined by measuring the uptake of 800 μM and 400 μM thymidine in the presence of increasing concentrations of inhibitor.

© 1999 Cancer Research Campaign

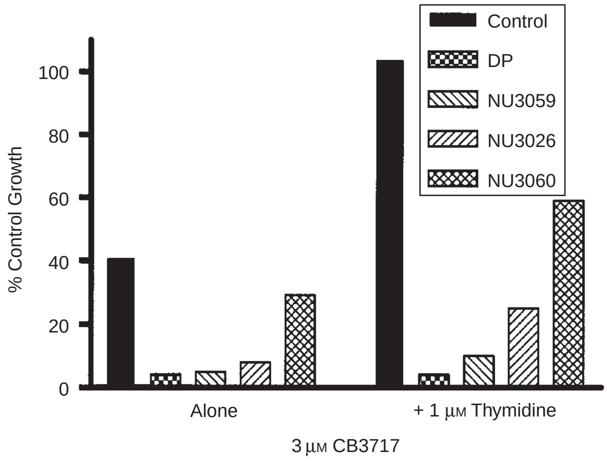

**Figure 1** Potentiation of CB3717 growth inhibition by dipyridamole and selected analogues. Cell growth was determined after 48-h exposure to 3 μM CB3717 with or without 1 μM thymidine supplementation in the presence or absence of 10 μM dipyridamole, NU3026, NU3059 or NU3060. Data, normalized by comparison with cell growth in the presence of 10 μM dipyridamole, NU3026, NU3059 or NU3060 alone, are the mean of three independent observations. Standard deviations (not shown) were < 15% in every case

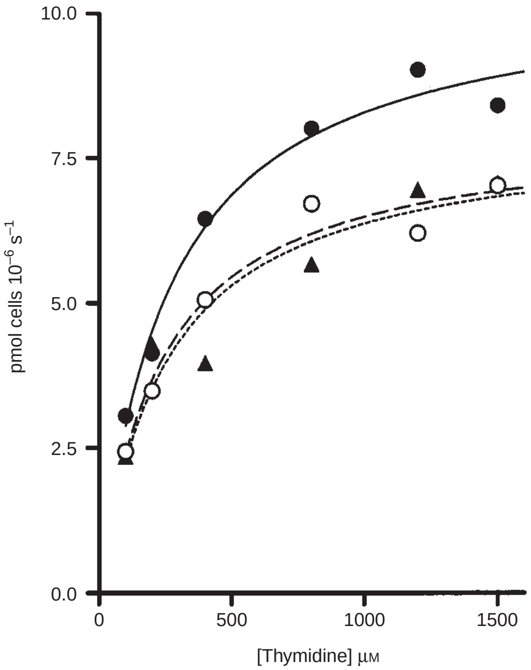

**Figure 2** Nucleoside transport inhibition by dipyridamole and NU3076. Thymidine uptake was measured in the absence of inhibitor (●, solid line), and in the presence of 0.3 μM dipyridamole, (○, dashed line) or 0.3 μM NU3076 (▲, dotted line). Lines were fitted as described in Materials and Methods

**Table 3** Inhibition of thymidine uptake by dipyridamole and NU3076 in the presence or absence of AGP

| | | Dipyridamole | NU3076 |
|---|---|---|---|
| Structure | |  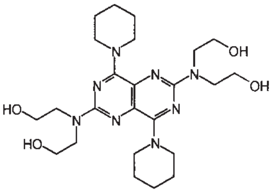 |  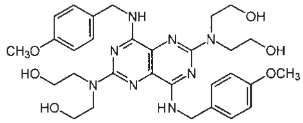 |
| $IC_{50}$ for thymidine uptake (μM) | | 0.36, 0.37 | 0.25 ± 0.08 (3) |
| Inhibitor concentration | 1 μM | 80 ± 8 (26)[a] | 56 ± 10 (7) |
| | 1 μM + AGP[b] | 4 ± 11 (9) | 46 ± 7 (7)[NS] |
| | 10 μM | 99 ± 2 (84) | 96 ± 8 (18) |
| | 10 μM + AGP | 13 ± 12 (27)[c] | 89 ± 14 (9)[NS] |

Figures are individual values or mean ± standard deviation with the number of observations given in parentheses. [a]Data are the % inhibition of thymidine uptake. [b]AGP concentration was 5 mg ml⁻¹. [c]Significantly different from value obtained in absence of AGP, $P < 0.01$ (unpaired Student's *t*-test). NS, not significantly different from value obtained in absence of AGP.

### Growth inhibition assays

L1210 cells in exponential growth were dispensed into each well of a 24-well plate at $10^4$ cells $ml^{-1}$, in RPMI-1640 medium supplemented with (10% v/v) dialysed serum containing CB3717, with or without thymidine and/or transport inhibitor (4–6 replicate wells for each drug combination). The DMSO concentration was controlled so as to be present at 1% (v/v) in the final incubation. Replicate aliquots of the remaining cell suspension were counted to obtain a time zero cell count. After incubation at 37°C for 48 h (to allow for a minimum of three control cell doublings) cells were counted using a Coulter Counter Model Z1 (Coulter Electronics, Luton, UK).

**Table 4** Kinetic constants for thymidine transport and inhibition by NU3076 and dipyridamole in L1210 cells

| | $K_t$ (μM) | $T_{max}$ (pmol/10⁻⁶ cells/s) | $T_{max}/K_t$ (pmol/10⁻⁶ cells/s/μM) |
|---|---|---|---|
| No inhibitor | 202 ± 35 (5) | 11 ± 1.1 (5) | 0.05 ± 0.01 (5) |
| 0.3 μM dipyridamole | 268 ± 66 (5)[a] | 8.4 ± 0.7 (5)[b] | 0.03 ± 0.01 (5)[b] |
| 0.3 μM NU3076 | 271 ± 61 (4)[a] | 8.5 ± 0.8 (4)[b] | 0.03 ± 0.004 (4)[b] |

Figures are mean ± s.d. for the number of independent experiments given in parenthesis. Significant difference from control (unpaired, two-tailed, Student's *t*-test) are given by: [a]$P < 0.1$; [b]$P < 0.05$.

© 1999 Cancer Research Campaign

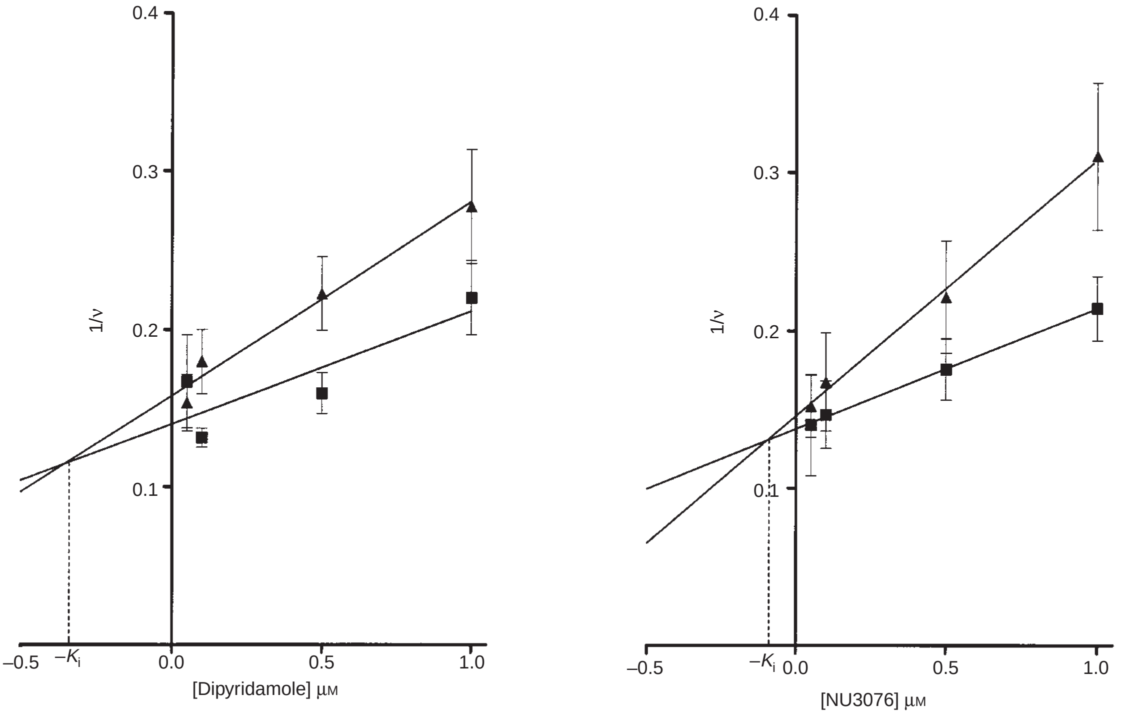

**Figure 3** Dixon plots used to determine the $K_i$ values for dipyridamole and NU3076-induced inhibition of thymidine uptake. The rate, υ, represents the rate of thymidine uptake (pmol/$10^{-6}$ cells/s) measured at 400 µM (▲) or 800 µM (■) thymidine. The data are the mean and standard deviation from three independent experiments and lines were fitted as described in Materials and Methods

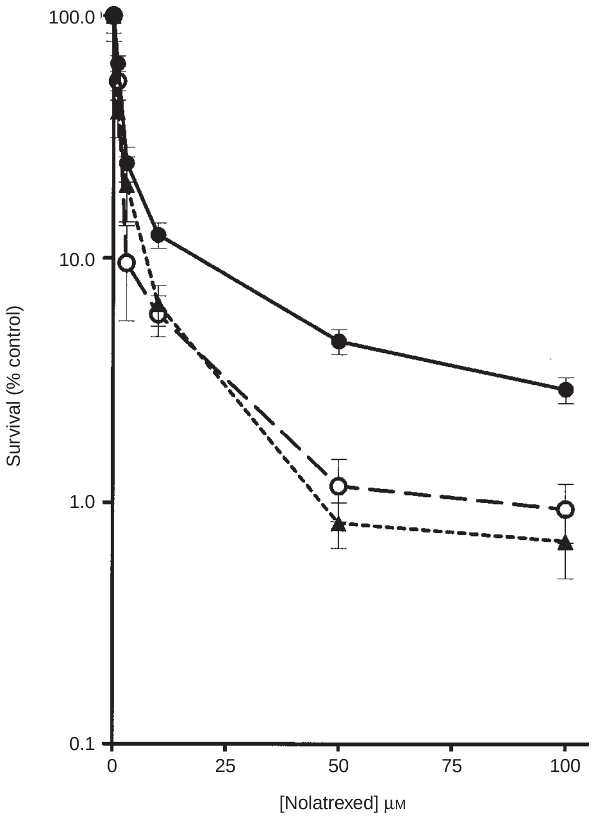

**Figure 4** Potentiation of nolatrexed cytotoxicity by 1 µM NU3076 and dipyridamole. Cells were exposed to nolatrexed alone (●), or in the presence of 1 µM NU3076 (▲) or 1 µM dipyridamole (○), for 16 h prior to seeding for colony formation in drug-free medium containing 0.125% (w/v) agarose. Data are mean ± s.d. of triplicate colony counts from each of the cell populations exposed in duplicate, from a single representative experiment

### Cytotoxicity assay

L1210 cells adapted to grow in dialysed serum were exposed to varying concentrations of nolatrexed or 5-FU in the presence or absence of 1 µM dipyridamole or 1 µM NU3076 for 16 h in medium containing (10% v/v) dialysed serum. The cells were resuspended in fresh medium to remove the drug, counted and seeded for colony formation in 0.125% (w/v) agarose (SeaKem, Flowgen, Sittingbourne, UK) in medium supplemented with 10% (v/v) undialysed serum. To investigate prevention of thymidine rescue, L1210 cells were incubated for 16 h with a fixed concentration of nolatrexed (0, 10 or 100 µM), with or without 1 µM thymidine, in the presence or absence of 1 µM dipyridamole or NU3076, and then counted and seeded for colony formation in 0.125% (w/v) agarose as above. The enhancement factor $EF_{90}$ was calculated using the following equation:

$$EF_{90} = LC_{90} \text{ nolatrexed alone}/LC_{90} \text{ nolatrexed + inhibitor}$$

(where $LC_{90}$ is the concentration of drug required to decrease cell survival by 90%). The enhancement factor at 50% survival, $EF_{50}$, was calculated by substituting $LC_{50}$ values in the above equation.

### RESULTS

### Dipyridamole analogues with modifications of the diethanolamine side chains

*Inhibition of thymidine uptake*

The inhibition of thymidine uptake by three novel compounds (NU3026, NU3059 and NU3060), with different substituents at the 2,6-position of the pyrimidopyrimidine ring was determined, and inhibition at concentrations of 1 and 10 µM of these inhibitors, as

 *© 1999 Cancer Research Campaign*

**Table 5** Potentiation of nolatrexed and 5-FU cytotoxicity by dipyridamole and NU3076

| Cytotoxic agent | Treatment | $LC_{50}$ μM | $LC_{90}$ μM | $EF_{50}$ | $EF_{90}$ |
|---|---|---|---|---|---|
| Nolatrexed | Control | 3.1 ± 1.9 | 22, 65 | | |
| Nolatrexed | 1 μM DP | 2.5 ± 1.7 | 10 ± 6 | 1.3 ± 0.1[a] | 7.5, 6.1 |
| Nolatrexed | 1 μM NU3076 | 2.1 ± 1.7 | 11 ± 3 | 1.6 ± 0.4[a] | 2.7, 5.9 |
| 5-FU | Control | 3.7 ± 1.5 | 20 ± 10 | | |
| 5-FU | 1 μM DP | 3.4 ± 1.0 | 19 ± 6 | 1.3 ± 0.3 | 1.2 ± 0.1 |
| 5-FU | 1 μM NU3076 | 4.1 ± 1.6 | 17 ± 9 | 1.1 ± 0.1 | 1.3 ± 0.2[a] |

The Enhancement Factors (EF) are the ratio of the $LC_{50}$ or $LC_{90}$ values for nolatrexed or 5-FU in the presence and absence of dipyridamole or NU3076. Results are expressed as individual values or mean ± s.d. of three experiments. [a]Indicates a significant difference from control by paired, two-tailed, Student's *t*-test.

well as the $IC_{50}$ values for inhibition of thymidine uptake, are given in Table 1. All three compounds were less potent than dipyridamole in the absence of AGP. However, in the presence of 5 mg $ml^{-1}$ AGP, dipyridamole was inactive as an inhibitor of thymidine uptake, whereas all of the novel compounds retained significant activity.

### *Potentiation of the growth inhibition produced by the TS inhibitor, CB3717*

The inhibition of the growth of L1210 cells by CB3717, with or without 1 μM thymidine, determined in the presence or absence of 10 μM nucleoside transport inhibitor is shown in Figure 1. All the inhibitors potentiated growth inhibition produced by 3 μM CB3717 in the absence of salvageable thymidine, and also prevented thymidine rescue from CB3717-induced growth inhibition. Comparable results were obtained by combining the transport inhibitors with 10 μM CB3717 (Table 2). Similarly, there was a modest potentiation of CB3717 growth inhibition by 1 μM dipyridamole, NU3059 and NU3026 and partial reversal of thymidine rescue by 1 μM dipyridamole and NU3059 (data not shown). For both potentiation of CB3717-induced growth inhibition and prevention of thymidine rescue the rank order of potency of the compounds was: dipyridamole > NU3059 > NU3026 > NU3060. Comparison of the inhibitory effect of 10 μM inhibitor on nucleoside transport (Table 1) with the effect on cell growth (in the presence of CB3717 + TdR) demonstrated a significant positive rank correlation (Spearman's rank correlation coefficient = 0.9, *P* = 0.083).

### A dipyridamole analogue modified at the 4,8-position (NU3076)

### *Inhibition of thymidine uptake in the presence and absence of AGP*

In an attempt to identify a potent dipyridamole analogue completely devoid of AGP binding, synthetic efforts were directed at replacing the 4,8-piperidino group of dipyridamole with other moieties. These modifications led to the identification of NU3076. As shown in Table 3, NU3076 was at least as potent as dipyridamole and AGP (5 mg $ml^{-1}$) did not significantly reduce the inhibition of thymidine uptake at either 10 μM or 1 μM NU3076 (equivalent to a 12- and 120-fold molar excess of AGP respectively). In contrast, AGP at this concentration abolished the inhibition of thymidine uptake by both 1 μM and 10 μM dipyridamole. NU3076 was therefore selected for further investigation and comparison with dipyridamole.

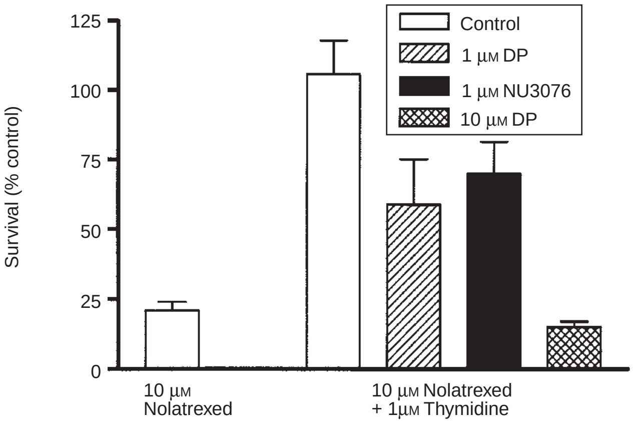

**Figure 5** Potentiation of 10 μM nolatrexed cytotoxicity and prevention of thymidine rescue by NU3076 and dipyridamole. Cells were exposed to 10 μM nolatrexed in the presence or absence of 1 μM thymidine (open bars), or 1 μM thymidine with 1 μM NU3076 (solid bars), 1 μM dipyridamole (hatched bars) or 10 μM dipyridamole (cross-hatched bars) for 16 h prior to seeding for colony formation in drug-free medium containing 0.125% (w/v) agarose. Data are mean ± s.d., of triplicate colony counts from each of the cell populations exposed in duplicate, from a single representative experiment

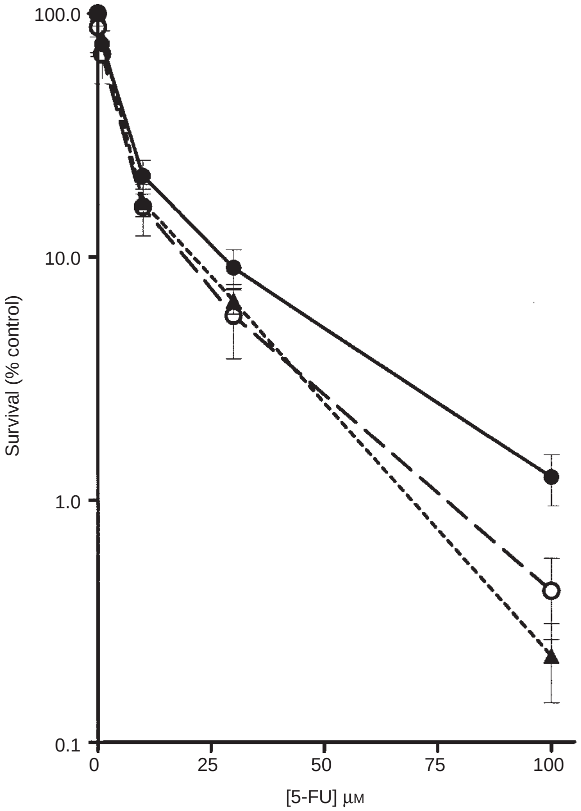

**Figure 6** Potentiation of 5-FU cytotoxicity by 1 μM NU3076 and dipyridamole. Cells were exposed to 5-FU alone (●), or 5-FU in the presence of 1 μM NU3076 (▲) or 1 μM dipyridamole (○) for 16 h prior to seeding for colony formation in drug-free medium containing 0.125% (w/v) agarose. Data are mean ± s.d. of triplicate colony counts from each of the cell populations exposed in duplicate, from a single representative experiment

*© 1999 Cancer Research Campaign*

### Inhibition kinetics

Kinetic measurements were performed to determine the mechanism responsible for the inhibition of thymidine uptake by dipyridamole and NU3076. The apparent $K_t$ and $T_{max}$ of nucleoside transport in L1210 cells was calculated in the absence and in the presence of 0.3 μM dipyridamole or 0.3 μM NU3076, Figure 2 and Table 4. There was a significant decrease in the apparent $T_{max}$ for thymidine transport in the presence of 0.3 μM dipyridamole and NU3076 ($P$ = 0.006 and $P$ = 0.015 respectively). The apparent $K_t$ values were only marginally different from the control value ($P$ = 0.083 and 0.069 for 0.3 μM dipyridamole and NU3076 respectively). When compared to control values, a significant decrease in the $T_{max}/K_t$ ratio was observed with both dipyridamole and NU3076, indicative (together with a decrease in $T_{max}$) of mixed inhibition. The $K_i$ values for dipyridamole and NU3076 were determined, using Dixon plots at thymidine concentrations of 400 and 800 μM, the $K_i$ for dipyridamole and NU3076 were estimated to be 0.28 μM and 0.1 μM respectively (Figure 3).

### Potentiation of TS inhibitor cytotoxicity

Neither 1 μM NU3076 nor 1 μM dipyridamole were cytotoxic per se against L1210 cells (cell survival ± standard error of 100 ± 24% and 100 ± 19% respectively). The clonogenic survival of L1210 cells exposed to varying concentrations of nolatrexed in the absence of thymidine, and in the presence or absence of 1 μM of dipyridamole or NU3076 is shown in Figure 4, corresponding $LC_{50}$ and $LC_{90}$ values are given in Table 5. NU3076 and dipyridamole at 1 μM produced equivalent potentiation of nolatrexed cytotoxicity (Figure 4 and Table 5) and, as cells were exposed to nolatrexed in medium supplemented with dialysed serum, potentiation by dipyridamole and NU3076 was not due to the prevention of thymidine rescue. Thymidine rescue from nolatrexed cytotoxicity in L1210 cells in the presence and absence of dipyridamole and NU3076 was also investigated. The cytotoxic effect of 10 μM nolatrexed was completely prevented by supplementing the medium with 1 μM thymidine and both dipyridamole and NU3076 at 1 μM were equi-active in preventing thymidine rescue, which was reduced but not blocked completely (Figure 5). In the presence of 10 μM dipyridamole thymidine rescue was completely blocked; the survival of cells exposed to nolatrexed and thymidine in the presence of 10 μM dipyridamole being equivalent to that of cells exposed to nolatrexed alone (Figure 5). It was not possible to investigate the prevention of thymidine rescue by 10 μM NU3076 due to the limited solubility of the analogue.

The effect of 1 μM dipyridamole or 1 μM NU3076 on the cytotoxicity of 5-FU was also determined. A representative survival curve is shown in Figure 6, and pooled $LC_{50}$ and $LC_{90}$ data (from three independent experiments) are given in Table 5. Significant potentiation of 5-FU cytotoxicity by dipyridamole and NU3076 was observed at high concentrations of 5-FU (30 μM and 100 μM) but at lower concentrations the potentiation was not significant. NU3076 also significantly reduced the $IC_{90}$ concentration of FU. These experiments were conducted in the absence of salvageable thymidine (dialysed serum) and hence potentiation was presumably due to inhibition of nucleoside efflux (deoxyuridine, fluorodeoxyuridine and/or fluorouridine) although this was not determined experimentally.

### DISCUSSION

Three dipyridamole analogues with altered 2,6-substituents (NU3026, NU3059 and NU3060) have been identified which, although less potent than dipyridamole in the absence of AGP, retain significant activity in the presence of this plasma protein. The three analogues were compared with dipyridamole as modulators of CB3717-induced growth inhibition and, in the absence of salvageable thymidine, all of the analogues increased the growth inhibitory effect of CB3717. In the absence of thymidine the potentiation of CB3717 activity was probably due to the inhibition of deoxuridine efflux, which has been shown in the case of dipyridamole to result in greater dUTP accumulation and DNA strand breakage (Curtin et al, 1991). All three dipyridamole analogues were also able to block thymidine rescue from CB3717-induced growth inhibition to a degree that was related to their potencies as inhibitors of nucleoside transport.

Substitution of the 4,8-piperidino groups of dipyridamole with a *p*-methoxybenzylamino group, as in the case of NU3076, resulted in both improved potency as a nucleoside transport inhibitor and reduced susceptibility to AGP binding. Notably, the activity of NU3076 was not significantly affected by a > 100-fold molar excess of AGP whereas the activity of dipyridamole was abolished under equivalent conditions. The kinetic parameters calculated in this study for thymidine uptake by L1210 cells, and the $K_i$ for dipyridamole are comparable to reported values (Plagemann and Wohlhueter, 1984). Both dipyridamole and NU3076 were found to exhibit mixed-type inhibition, indicating that the interaction with the nucleoside transporter is more complex than simple competition between dipyridamole and nucleosides for the substrate binding site, again in agreement with previous observations (Woffendin and Plagemann, 1987). Dipyridamole-sensitive nucleoside transport is mediated by equilibrative transporters, of which there are two subtypes (*es* and *ei*) which exhibit different kinetic properties (Jarvis, 1986; Plagemann et al, 1988; Hammond, 1991) and are the product of distinct genes (Griffiths et al, 1997; Crawford et al, 1998). The proportion of carriers expressed in the L1210 cells used in these studies was found to be about 70% es, and 30% *ei* (K Bowman, unpublished data), in agreement with published data (Belt, 1983). The affinity of dipyridamole for the es transporter is probably 2.5 × higher than for the *ei* transporter (Hammond, 1991), producing different inhibition constants for each transporter subtype. Thus, the mixed-type inhibition of thymidine uptake observed with dipyridamole and NU3076 in L1210 cells may reflect the presence of the two different transporter proteins.

Both nolatrexed and 5-FU were cytotoxic to L1210 cells in a concentration-dependent manner. The observation that 1 μM thymidine completely reversed the cytotoxic effects of nolatrexed in L1210 cells confirms that TS is the locus of action of the drug in these cells, as has been demonstrated in other cell lines (Webber et al, 1996). Cell survival decreased in response to increasing concentrations of nolatrexed, with only 10–20% of cells surviving exposure to 10 μM nolatrexed. At concentrations above 10 μM nolatrexed there was very little further increase in cytotoxicity, suggesting a fraction of the cells are resistant to inhibition of TS, probably because they failed to enter a nolatrexed-sensitive phase of the cell cycle during the period of drug exposure. Cell killing also increased in response to increasing 5-FU concentrations; however, in contrast to the pattern of cytotoxicity of nolatrexed, there was no apparent plateau in cell survival. The lack of a resistant fraction of cells may reflect the fact that 5-FU has a number of loci of action in addition to TS inhibition, e.g. FUTP and FdUTP incorporation into RNA and DNA respectively.

Both dipyridamole and the novel analogue NU3076 were approximately equipotent as enhancers of the cytotoxicity of nolatrexed and 5-FU and, since the clonogenic cell survival studies

 © 1999 Cancer Research Campaign were carried out in medium supplemented with dialysed serum, potentiation by dipyridamole and NU3076 was not due to inhibition of thymidine rescue. By analogy with previous studies (Grem and Fischer 1985, 1989; Curtin et al, 1988, 1991) potentiation in the absence of thymidine is most likely to be due to inhibition of nucleoside efflux (deoxuridine, fluorodeoxuridine, fluorouridine) leading to greater accumulation of cytotoxic nucleosides. Modulation of thymidine rescue from nolatrexed cytotoxicity by dipyridamole and NU3076 was also investigated, and the complete reversal of nolatrexed cytotoxicity by 1 μM thymidine was partially inhibited by both 1 μM dipyridamole and NU3076, and completely blocked by 10 μM dipyridamole. In nucleoside transport assays thymidine uptake was inhibited by 80% at 1 μM dipyridamole and by 56% at 1 μM NU3076, consistent with the partial rescue from nolatrexed cytotoxicity at this concentration. In the presence of 10 μM dipyridamole, which produced 99% inhibition of thymidine uptake in transport studies, thymidine rescue was completely blocked such that the survival of cells exposed to nolatrexed, thymidine and 10 μM dipyridamole was not significantly different from that of cells exposed to nolatrexed alone. It was not possible to investigate the prevention of thymidine rescue by 10 μM NU3076 due to the limited solubility of this compound. Recent data from this laboratory (P Smith, manuscript in preparation) indicate that NU3076 can block thymidine rescue from TS inhibitor cytotoxicity in human cancer cell lines.

Both dipyridamole and NU3076 potentiated 5-FU cytotoxicity to a similar extent. However, potentiation was only apparent at high concentrations of 5-FU, and the degree of potentiation of 5-FU was less than that observed for nolatrexed. In other studies dipyridamole has been shown to potentiate 5-FU cytotoxicity by different mechanisms in different cell lines. In WiDr human colon adenocarcinoma cells, dipyridamole potentiated 5-FU cytotoxicity by depletion of cellular dTTP pools (Lonn et al, 1989). In HCT 116 cells, the potentiation of 5-FU by dipyridamole was considered to be a consequence of both elevated dUMP levels and increased intracellular accumulation of FdUMP, the latter formed from FUdR. These nucleotide pool changes were thought to reflect the combined effects of de-repression of dCMP deaminase and thymidine kinase, following loss of feedback inhibition by dTTP, and inhibition of deoxyuridine and fluorodeoxuridine efflux (Grem and Fisher, 1985, 1989). An investigation into which of these mechanisms is critical to 5-FU cytotoxicity in L1210 cells has not been undertaken, but clearly TS inhibition is not the only important locus of action of the drug.

From the data reported here it is apparent that AGP binding, which has limited the clinical use of dipyridamole as an enhancer of antimetabolite chemotherapy, can be overcome. Potentiation of the growth inhibition and cytotoxicity of TS inhibitors by novel dipyridamole analogues was related to nucleoside transport inhibitory potency. All of the compounds described here were more potent than dipyridamole in the presence of physiological concentrations of AGP, and the potency of one compound, NU3076, was not significantly affected by AGP binding.

### ACKNOWLEDGEMENTS

We gratefully acknowledge the support of the North of England Cancer Research Campaign, the Cancer Research Campaign and Cancer Research Campaign Technology.

### REFERENCES

Belt JA (1983) Heterogeneity of nucleoside transport in mammalian cells two types of transport activity in L1210 and other cultured neoplastic cells. *Mol Pharmacol* **24**: 479–484

Budd GT, Jayaraj A, Grabowski D, Adelstein D, Bauer L, Boyett J, Bukowski R, Murthy S and Weick J (1990) Phase I trial of dipyridamole with 5-fluorouracil and folinic acid. *Cancer Res* **50**: 7206–7211

Calvert AH, Alison DL, Harland SJ, Jackman AL, Jones TR, Newell DR, Siddik ZH, Wiltshaw E, McElwain TJ, Smith IE and Harrap KR (1986) A phase I evaluation of the quinazoline antifolate thymidylate synthase inhibitor $N^{10}$-propargyl-5,8-dideazafolic acid, CB3717. *J Am Soc Clin Oncol* **4**: 1245–1252

Chen TR (1977) In situ detection of mycoplasma contamination in cell cultures by fluorescent Hoechst 33258 stain. *Exp Cell Res* **104**: 255–262

Crawford CR, Ng CYC, Noel D and Belt JA (1990) Nucleoside transport in L1210 murine leukemia cells. *J Biol Chem* **265**: 9732–9736

Crawford CR, Patel DH, Naeve C and Belt JA (1998) Cloning of the human equilibrative, nitrobenzylmercaptopurine riboside (NBMPR)-insensitive nucleoside transporter *ei* by functional expression in a transport-deficient cell line. *J Biol Chem* **273**: 5288–5293

Curtin NJ, Newell DR and Harris AL (1989) Modulation of dipyridamole action by $\alpha_1$-acid glycoprotein. *Biochem Pharmacol* **38**: 3281–3288

Curtin NJ, Harris AL and Aherne GW (1991) Mechanism of cell death following thymidylate synthase inhibition: 2′-deoxyuridine-5′-triphosphate accumulation, DNA damage and growth inhibition following exposure to CB3717 and dipyridamole. *Cancer Res* **51**: 2346–2352

Fox, M, Boyle, JM and Kinsella, AR (1991) Nucleoside salvage and resistance to antimetabolite anticancer agents. *Br J Cancer* **64**: 428–436

Goel R and Howell SB (1992) Modulation of the activity of cancer chemotherapeutic agents by dipyridamole. In: *New Drugs, Concepts and Results in Cancer Chemotherapy,* Muggia FM (ed), pp. 19–44. Kluwer: Amsterdam

Grem JL and Fischer PH (1985) Augmentation of 5-fluorouracil cytotoxicity in human colon cancer cells by dipyridamole. *Cancer Res* **45**: 2967–2972

Grem JL and Fischer PH (1989) Enhancement of 5-fluorouracil anticancer activity by dipyridamole. *Pharmacol Ther* **40**: 349–379

Griffiths M, Beaumont N, Yao SYM, Sundaram M, Boumah CE, Davies A, Kwong FYP, Coe I, Cass CE, Young JD and Baldwin SA (1997) Cloning of a human nucleoside transporter implicated in the cellular uptake of adenosine and chemotherapeutic drugs. *Nat Med* **3**: 89–93

Hammond JR (1991) Comparative pharmacology of the nitrobenzylthioguanosine-sensitive and -resistant nucleoside transport mechanisms of Ehrlich ascites tumour cells. *J Pharmacol Exp Ther* **259**: 799–807

Jackman AL, Taylor GA, Calvert AH and Harrap KR (1984) Modulation of antimetabolite effects: effects of thymidine on the efficacy of quinazoline-based thymidylate synthetase inhibitor CB3717. *Biochem Pharmacol* **33**: 3269–3275

Jarvis SM (1986) Nitrobenzylthioinosine-sensitive nucleoside transport system: mechanism of inhibition by dipyridamole. *Mol Pharmacol* **30**: 659–665

Jones TR, Calvert AH, Jackman AL, Brown SJ, Jones M, and Harrap KR (1981) A potent antitumour quinazoline inhibitor of thymidylate synthetase: synthesis, biological properties and therapeutic results in mice. *Eur J Cancer* **17**: 11–19

Kinsella, AR and Harran, MS (1991) Decreasing sensitivity to cytotoxic agents parallels increasing tumorigenicity in human fibroblasts. *Cancer Res* **51**: 1855–1859

Köhne-Wömpner C-H, Schmoll HJ, Wilke H, Schöber C, Bodenstein H, Gropp C, Hiddermann W, Kunth A, Schmitz-Hübner U and Weiß J (1989) Comparative activity of 5-FU/high dose folinic acid ± dipyridamole – a randomised multi center phase II trial in advanced colorectal cancer. *Proc Eur Conf Clin Oncol* **5**: 703

Kremer JMH, Wilting J and Janssen LHM (1988) Drug binding to human alpha-1-acid glycoprotein in health and disease. *Pharmacol Rev* **40**: 1–47

Lonn U, Lonn S, Nylen U and Winblad G (1989) 5-Fluoropyrimidine-induced DNA damage in human colon adenocarcinoma and its augmentation by the nucleoside transport inhibitor dipyridamole. *Cancer Res* **49**: 1085–1089

Mahony C, Wolfram KM, Coccetto DM and Bjornsson TD (1982) Dipyridamole kinetics. *Clin Pharm Ther* **31**: 330–338

Plagemann PGW and Wohlhueter RM (1984) Nucleoside transport in cultured mammalian cells. Multiple forms with different sensitivity to inhibition by nitrobenzylthioinosine or hypoxanthine. *Biochim Biophys Acta* **773**: 39–52

Rustum YM, Harstrick A, Cao S, Vanhoefer U, Yin M-B, Wilke H and Seeber S (1997) Thymidylate synthase inhibitors in cancer therapy: direct and indirect inhibitors. *J Clin Oncol* **15**: 389–400

© 1999 Cancer Research Campaign 

Plagemann PGW, Wohlhueter RM and Woffendin C (1988) Nucleoside and nucleobase transport in animal cells. *Biochim Biophys Acta* **947**: 405–443
Schilsky RL (1992) Antimetabolities. In: *The Chemotherapy Source Book*. Perry MC (ed), pp 301–317. Williams and Wilkins: Baltimore
Schmoll H-J, Harstrick A, Köhne-Wömpner C-H, Schöber C, Wilke H and Poliwoda H (1990) Modulation of cytotoxic drug activity by dipyridamole. *Cancer Treat Rev* **17**: 57–65
Wadler S, Subar M, Green MD, Wiernik PH and Muggia FM (1987) Phase II trial of oral methotrexate and dipyridamole in colorectal carcinoma. *Cancer Treat Rep* **71**: 821–824
Webber S, Bartlett CA, Boritzki TJ, Hillard JA, Howland EF, Johnston AL, Kosa M, Margosiak SA, Morse CA and Shetty BV (1996) AG337, a novel lipophilic thymidylate synthase inhibitor: in vitro and in vivo preclinical studies. *Cancer Chemother Pharmacol* **37**: 509–517
Weber G (1983) Biochemical strategy of cancer cells and the design of chemotherapy: GHA Clowes Memorial Lecture. *Cancer Res* **43**: 3466–3492
Willson JKV, Fischer PH, Tutsch K, Albarti D, Simnom K, Hamilton RD, Bruggink J, Koeller JM, Tormey DC, Earhart RH, Rachosky A and Trump DL (1988) Phase I clinical trial of a combination of dipyridamole and acivicin based upon inhibition of nucleoside salvage. *Cancer Res* **48**: 5585–5590
Woffendin C and Plagemann PGW (1987) Interaction of [$^3$H]dipyridamole with the nucleoside transporters of human erythrocytes and cultured animal cells. *J Membrance Biol* **98**: 189–100
Wohlhueter RM, Marz R, Graff JC and Plagemann PGW (1978) A rapid-mixing technique to measure transport in suspended animal cells: applications to nucleoside transport in Novikoff rat hepatoma cells. *Methods Cell Biol* **20**: 211–236

 *© 1999 Cancer Research Campaign*
